# Rigosertib and Cholangiocarcinoma: A Cell Cycle Affair

**DOI:** 10.3390/ijms23010213

**Published:** 2021-12-25

**Authors:** Alessio Malacrida, Guido Cavaletti, Mariarosaria Miloso

**Affiliations:** Experimental Neurology Unit, School of Medicine and Surgery, University of Milano-Bicocca, Via Cadore 48, 20900 Monza, MB, Italy; guido.cavaletti@unimib.it (G.C.); mariarosaria.miloso@unimib.it (M.M.)

**Keywords:** Rigosertib, PLK1 inhibitor, cholangiocarcinoma, EGI-1 cells, cell cycle, p53, Cyclin B, CDK1, EMI1

## Abstract

Rigosertib is multi-kinase inhibitor that could represent an interesting therapeutic option for non-resectable patients with cholangiocarcinoma, a very aggressive hepatic cancer with limited effective treatments. The Western blotting technique was used to evaluate alterations in the expression of proteins involved in the regulation of the cell cycle of cholangiocarcinoma EGI-1 cells. Our results show an increase in EMI1 and Cyclin B protein levels after Rigosertib treatment. Moreover, the phosphorylation of CDK1 is significantly reduced by Rigosertib, while PLK1 expression increased after 24 h of treatment and decreased after 48 h. Finally, we evaluated the role of p53. Its levels increase after Rig treatment, and, as shown in the cell viability experiment with the p53 inhibitor Pifithrin, its activity is necessary for the effects of Rigosertib against the cell viability of EGI-1 cells. In conclusion, we hypothesized the mechanism of the action of Rigosertib against cholangiocarcinoma EGI-1 cells, highlighting the importance of proteins involved in the regulation of cell cycles. The CDK1-Cyclin B complex and p53 play an important role, explaining the Block in the G2/M phase of the cell cycle and the effect on cell viability

## 1. Introduction

Cholangiocarcinoma (CCA) is the second most common hepatic cancer after hepatocellular carcinoma [1]. It is very aggressive and heterogeneous, with an increasing incidence and mortality rate worldwide [1,2]. CCA is considered an orphan tumor, due to limited therapeutic options [3,4,5]. Current chemotherapy for CCA includes the combination of Gemcitabine with platin-based drugs, but the response is often unsuccessful [5]. In our previous article [6], we have demonstrated that Rigosertib (Rig), a non-ATP competitive multi-kinase inhibitor [7], could represent an interesting therapeutic option for non-resectable patients with CCA. Rig was able not only to impair EGI-1 CCA cell viability, but also other cellular processes, demonstrating that the Rig mechanism of the action is complex.

In the present study we have analyzed the possible molecular mechanisms responsible for Rig effects on EGI-1 cell cycle and mitotic failure, demonstrating the fundamental role played by the Cyclin B-CDK1 complex and p53 in the Rig effect.

## 2. Results and Discussion

### 2.1. EMI1 and Cyclin B Role in Rig Effect against EGI-1 Cells

**Figure 1:**
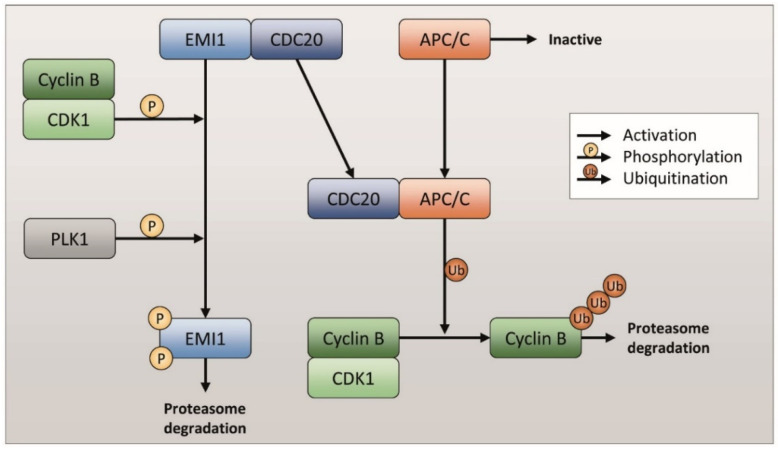
Cyclin B ubiquitination and proteasome degradation pathway.

EMI1 (early mitotic inhibitor) is a protein involved in the regulation of mitosis thanks to its indirect inhibition of APC/C [8,9]. Indeed, in physiological conditions, EMI1 keeps CDC20 inactive thanks to a direct interaction. When EMI1 is phosphorylated both by the Cyclin B-CDK1 complex and PLK1, it is subsequently polyubiquitinated by SCF and degraded by the proteasome. Both phosphorylations are required for EMI1 polyubiquitination and degradation. EMI1 degradation causes the release of CDC20, which is associated with APC/C. The complex CDC20-APC/C is therefore responsible for the polyubiquitination and subsequent degradation of Cyclin B (Figure 1) [10,11]. This event is necessary for CDK1 kinase inactivation and the exit from mitosis [12,13].

**Figure 2:**
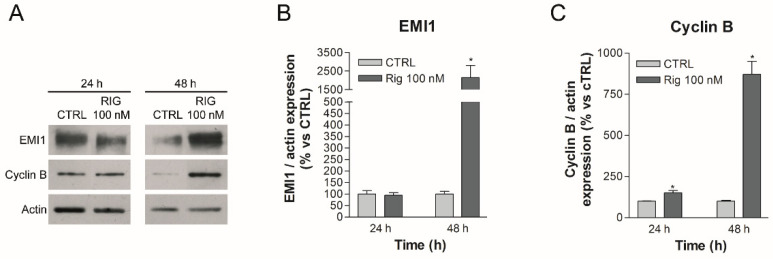
Western blot analysis of EMI1 and cyclin B in EGI-1 cells treated with Rig. (**A**) Representative images of EMI1, Cyclin B and actin after treatment with 100 nM Rig for 24 and 48 h. (**B**) The graph represents the quantification of EMI1 western blot, normalized to actin. (**C**) The graph represents the quantification of cyclin B western blot, normalized to actin. All the graphs are represented as the mean percentage ± SD of at least three independent experiments and are compared to untreated controls arbitrarily set to 100%. * *p* < 0.05 vs. CTRL.

As reported in the literature, Rig inhibits PLK1, making it unable to phosphorylate EMI1 [14,15]. In this way, one of the two fundamental phosphorylations of EMI1 is missing. EMI1 is therefore not degraded by proteasome and accumulates inside the cell. On the basis of the scheme reported in Figure 1, we have evaluated whether Rig treatment of EGI-1 cells is able to induce changes in the pathway described above. As shown in Figure 2, in EGI-1 cells after 24 h of treatment, Rig 100 nM was not able to alter EMI1 protein expression, but, after 48 h, Rig induced a significant increase in EMI1 protein levels (Figure 2A,B). Furthermore, after 24 and 48 h of treatment with Rig 100 nM (Figure 2A,C), levels of Cyclin B rose significantly, with a very relevant increase after 48 h of treatment.

### 2.2. CDK1 and PLK1 Role in Rig Effect against EGI-1 Cells

**Figure 3:**
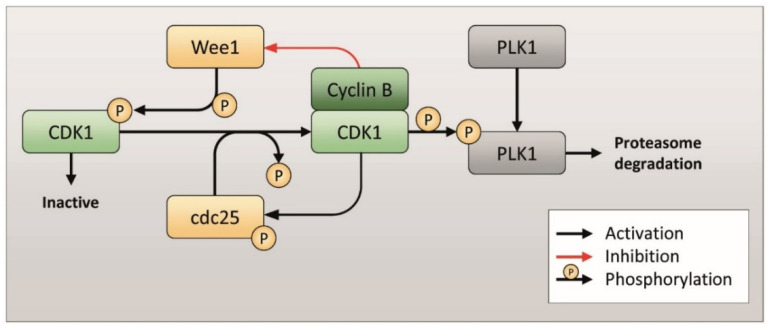
PLK1 phosphorylation and proteasome degradation pathway.

The Cyclin B-CDK1 complex is an important player in the regulation of the G2/M phase of the cell cycle. The complex is responsible for the phosphorylation and activation of downstream protein kinases and several structural proteins involved in mitotic spindle formation, cell architecture reorganization and cytokinesis during mitosis [16,17]. In physiological conditions, CDK1 is phosphorylated and inactive. In the G2/M phase, the increase of Cyclin B expression and the activation of Cyclin B-CDK1 complex induces the dephosphorylation of CDK1 by cdc25 and the inhibition of Wee1 (a phosphorylase responsible of CDK1 phosphorylation) [18,19,20]. Moreover, the active Cyclin B-CDK1 complex phosphorylates PLK1, inducing its degradation by proteasome (Figure 3) [21,22,23].

**Figure 4:**
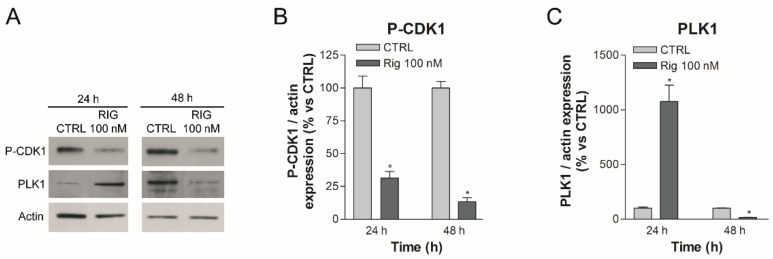
Western blot analysis of P-CDK1 and PLK1 in EGI-1 cells treated with Rig. (**A**) Representative images of P-CDK1, PLK1 and actin after treatment with different concentrations of 100 nM Rig for 24 and 48 h. (**B**) The graph represents the quantification of P-CDK1 Western blot, normalized to actin. (**C**) The graph represents the quantification of PLK1 western blot, normalized to actin. All the graphs are represented as the mean percentage ± SD of at least three independent experiments and are compared to untreated controls arbitrarily set to 100%. * *p* < 0.05 vs. CTRL.

In EGI-1 cells after Rig treatment, the observed accumulation of Cyclin B could lead to a constant activation of the Cyclin B-CDK1 complex. This complex feeds itself: it inhibits Wee1, responsible for the phosphorylation and inactivation of CDK1, and at the same time activates cdc25, responsible for the dephosphorylation and activation of CDK1 [24]. In our western blot experiments, we demonstrated that, in EGI-1 cells, Rig 100 nM reduced CDK1 phosphorylation both after 24 and 48 h of treatment (Figure 4A,B). Conversely, in EGI-1 cells we observed a significant increase in CDK1 total form in the presence of Rig. (Supplementary Figure S1). Lee and collaborators demonstrated that CDK1 is degraded by proteasome [25], suggesting that the inhibition of proteasome activity induced by Rig [6] could be responsible for the increase in CDK1 level observed. On the basis of our results, we propose that the Cyclin B-CDK1 complex is therefore overexpressed (Cyclin B and CDK1) and hyper-activated (CDK1).

The expression of PLK1 in physiological conditions is dependent on the phase of the cell cycle: its expression begins to increase in the G2 phase and before reaching a peak in the M phase [26]. In our previous article, we have shown that Rig induced an arrest in the G2/M phase at 24 h of treatment (about 50% cells were in G2/M phase) [6]. Therefore, in this state, the mechanisms that lead to the expression of cell cycle-dependent PLK1 remain active and PLK1 is expressed excessively, as we demonstrated by Western blotting after 24 h of treatment with Rig 100 nM (Figure 4A,C). However, PLK1 should be kept inhibited by Rig. Subsequently, the accumulation of Cyclin B observed previously and the hyperactivation of CDK1 lead to the phosphorylation and consequent degradation of PLK1. This, combined with the reduced gene expression of PLK1 induced by p53 (as we will discuss in the next paragraph), leads to a significant reduction of PLK1, as we demonstrated by Western blotting after 48 h of treatment with Rig 100 nM (Figure 4A,C). This important reduction of PLK1, coupled with its direct inhibition induced by Rig, could also explain the effect observed on proteasome and autophagy in our previous work [6]. In fact, in the literature it is reported that PLK1 inactivation causes a significant increase in autophagic activity through the inhibition of the mTOR pathway [27]. Moreover, α3-subunits of proteasome can be phosphorylated by PLK1, increasing the chymotrypsin-like activity [28].

### 2.3. p53 Role in Rig Effect against EGI-1 Cells

**Figure 5:**
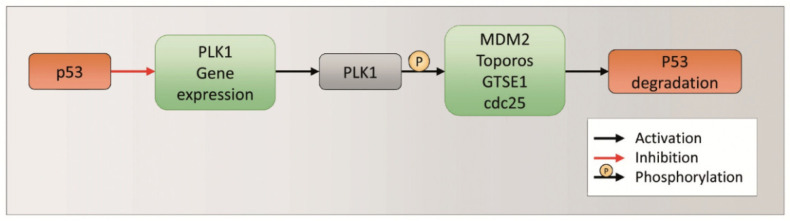
p53 and PLK1 expression and degradation regulation.

p53 is a tumor suppressor protein involved in the regulation of cell growth arrest and apoptosis related genes, in response to stress signals. It thereby influences cell death, cell-cycle regulation and DNA repair [29,30,31]. Furthermore, p53 is closely related to PLK1, as these two proteins indirectly regulate each other’s expression and degradation. Moreover, in greater detail, when p53 is activated, it negatively regulates the gene expression of PLK1 through different mechanisms (p21, miRNA, FoxM1, PLK1 gene promoter) [32]. When p53 is not activated and/or not expressed, PLK1 is normally expressed and activated. On the other hand, active PLK1 regulates the degradation of p53 through phosphorylation of various targets (MDM2, Toporos, GTSE1, cdc25) (Figure 5) [32].

**Figure 6:**
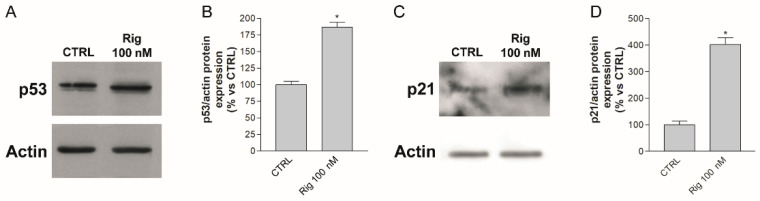
p53 and p21 expression in EGI-1 cells treated with Rig. (**A**) Representative image of p53 and actin Western blot after 24 h of treatment with Rig 100 nM. (**B**) The graph represents the quantification of p53 Western blot and data are represented as the mean percentage ± SD of p53 normalized to actin of three independent experiments, compared to untreated controls arbitrarily set to 100%. (**C**) Representative image of p21 and actin Western blot after 24 h of treatment with Rig 100 nM. (**D**) The graph represents the quantification of p21 Western blot and data are represented as the mean percentage ± SD of p21 normalized to actin of three independent experiments, compared to untreated controls arbitrarily set to 100%. * *p* < 0.05 vs. CTRL.

In the presence of Rig, the inhibition of PLK1 should not allow the phosphorylation and activation of MDM2, Toporos, GTSE1, cdc25. Consequently, these proteins could not induce the degradation of p53, which accumulates. In order to demonstrate this hypothesis, we performed Western blot experiments to evaluate the p53 level. After 24 h of EGI-1 treatment with Rig 100 nM, we observed a significant increase in p53 compared to CTRL (Figure 6A,B). Furthermore, we analyzed the level of p21, which is not only a p53 target, but is also able to negatively regulate the gene expression of PLK1. The treatment of EGI-1 cells with Rig 100 nm for 24 h induced a significant increase in p21 level compared to CTRL. Our results suggest that Rig, inducing p53 protein accumulation (Figure 6A,B), caused the increase in p21 protein (Figure 6C,D), responsible in turn for the downregulation of PLK1 (Figure 4A,C).

### 2.4. p53 Role in Rig Effect on Cell Viability of EGI-1 Cells

**Figure 7:**
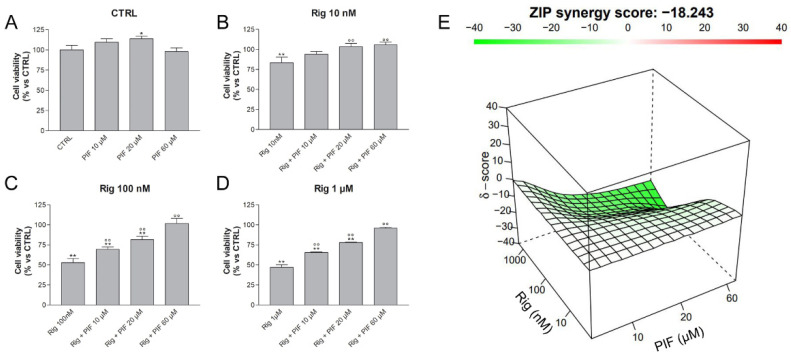
Cell viability of EGI-1 cells treated with different combinations of Rig and PIF. EGI-1 cells were treated for 24 h with different concentrations of PIF (0, 10, 20, 60 µM), alone (**A**) or in combination with Rig 10 nM (**B**), Rig 100 nM (**C**) and Rig 1 µM (**D**). Graphs are represented as the mean percentage ± SD compared to untreated control (CTRL), arbitrarily set to 100%, from at least three independent experiments. The antagonism of Rig and PIF combinations is calculated by the Synergy Finder web application and is represented in graph E: a green color indicates an antagonistic effect, a white color an additive effect, and a red color a synergistic effect. * *p* < 0.05 and ** *p* < 0.01 vs. CTRL; °° *p* < 0.01 vs. respective Rig.

To evaluate whether the observed alteration of p53 protein expression was involved in the effect of Rig on EGI-1 cell viability, cells were treated with Rig (10 nM, 100 nM and 1 µM) and with different concentrations of Cyclic Pifithrin alpha (PIF) (10, 20, 60 µM), a reversible inhibitor of p53-mediated apoptosis and p53-dependent gene transcription. Cell viability was assessed with MTT assay (Figure 7). PIF alone had no effect on cell viability and only a slight increase was observed (Figure 7A). 10 nM Rig alone reduced cell viability, while in combination with PIF, its effect was almost completely reverted by all concentrations of PIF evaluated (Figure 7B). Both Rig 100 nM and 1 µM reduced the cell viability of EGI-1 cells by about 50%. However, this effect was modified in a dose-dependent manner by PIF treatment and, in the presence of 60 µM PIF, with both Rig concentrations cell viability was almost comparable to untreated controls (Figure 7C,D). Our results demonstrated that the reduction of the cell viability of EGI-1 cells induced by Rig is p53 dependent.

Indeed, synergism/antagonism analysis with Synergy Finder web application [33] provided a combination score between Rig and PIF of −18.243, indicative of an antagonistic effect of PIF on Rig (Figure 7E). This result corroborates the role of p53 in Rig action.

## 3. Conclusions

In this work we demonstrated that Rig modulates the level of expression of several key proteins involved in cell-cycle regulation, such as PLK1, EMI1, CDK1 and Cyclin B. In our previous article [6], we observed that after 24 h of Rig treatment EGI-1 CCA, cells are blocked in the G2/M phase of the cell cycle. Subsequently, at 48 h of Rig treatment, the percentage of EGI-1 cells blocked in G2/M decreased; meanwhile, a significant increase in the percentage of polyploid cells was observed. The results presented in the current work could explain how Rig affects the cell cycle. The block in G2/M of EGI-1 cells treated for 24 h with Rig could be due to the increase in PLK1 protein expression and CDK1 activation. Subsequently, after 48 h of Rig treatment, the lack of degradation of cyclin B and therefore the lack of inactivation of its complex with CDK-1 could result in the persistence of the block in G2/M, inducing aberrant mitosis and the increase of nuclear DNA content with the appearance of polyploid cells. However, Rig could also act on the cell cycle through other types of mechanisms, such as microtubules destabilization [15], though in this manuscript we have focused our attention on PLK1, considered the main target of Rig. Finally, the increase in p53 level induced by Rig could also further induce the reduction of the expression of PLK1 and the maintenance of the active CDK1-Cyclin B complex, causing persistence in the block in G2/M.

In conclusion, the CDK1-Cyclin B complex plays an important role in the mechanism of the action of Rig in EGI-1 CCA cells. Further studies will be necessary to deepen the complex mechanism of the action of Rig in order to optimize its use in clinical settings.

## 4. Materials and Methods

### 4.1. Cell Cultures and Reagents

Human cholangiocarcinoma EGI-1 (DSMZ-German Collection of Microorganisms and Cell Cultures) cells were cultured in RPMI 1640 medium supplemented with 10% fetal bovine serum (FBS), 1% L-glutamine, 1% penicillin and streptomycin (Euroclone, Pero, Italy).

Rigosertib (Rig), kindly provided by Onconova Therapeutics (Newtown, PA, USA), was resuspended in water at 105 mM concentration and then diluted to working concentrations directly in the culture medium.

Cyclic Pifithrin alpha (PIF) (Prodotti Gianni, Milano, Italy) was resuspended in DMSO at 10 mM concentration and then diluted to a working concentration directly in the culture medium.

### 4.2. Western Blotting

Cells were seeded in 6 well plates at 250 × 103 cells/well density and were treated with Rig. Cells were seeded and treated as described in the trypan blue vital count paragraph. After incubation with the drugs, cells were lysed with a lysis buffer (5 mM Hepes pH 7.5, 150 mM NaCl, 10% Glycerol, 1% Triton X100, 1.5 mM MgCl2, 5 mM EGTA, 0.1 M PMSF, 1% aprotinine, 0.1 M Na-pyrophosphate, 0.5 M Na3VO4). Protein content was quantified using the Bradford method. 10 µg of proteins were separated in SDS-PAGE gel and transferred to a nitrocellulose membrane. Western blotting against p53 (1:1000, #9282, Cell Signaling, Danvers, MA, USA), EMI1 (1:1000, #sc-365212, Santa Cruz Biotechnology, Dallas, TX, USA), Cyclin B (1:1000, #sc-166210, Santa Cruz Biotechnology, Dallas, TX, USA), P-CDK1 (1:1000, #9111, Cell Signaling, Danvers, MA, USA), CDK1 (1:1000, #MA5-15629, Invitrogen, Waltham, MA, USA), and PLK1 (1:1000, #sc-17783, Santa Cruz Biotechnology, Dallas, TX, USA) and p21 (1:1000, #2947, Cell Signaling, Danvers, MA, USA) was performed following the manufacturer’s instructions. Immunoreactive proteins were visualized using an ECL chemiluminescence system (Amersham, Marlborough, MA, USA) and bands were quantified using ImageJ 1.52p software.

### 4.3. MTT Assay

Cells were seeded in 96 well plates at 10 × 10^3^ cells/well density and were treated with Rig, PIF or a combination of the two compounds. After 24 h, an MTT assay was performed to assess cell viability. Briefly, a 0.5 mg/mL solution of MTT was added to each well, and after 4 h formazan crystals were resuspended in acidified 2-propanol. Absorbance was then measured using a microplate reader at 570 nm (BMG Labtech, Ortenberg, Germany).

To evaluate the synergistic/antagonistic effect of PIF on Rig, we used the Synergy Finder web application and calculated the zip synergy score [33]. A score lower than −10 is indicative of an antagonistic effect (the green color in the graph), between −10 and +10 of an additive effect (the white color in the graph), and higher than +10 of a synergistic effect (the red color in the graph).

### 4.4. Statistical Analysis

Data are reported as mean ± standard deviation (SD) from at least three independent experiments. Statistical analysis was performed using GraphPad Prism 3 software. The differences between control and treated cells were evaluated using the Student’s *t*-test or One Way ANOVA analysis of variance, followed by Dunnet’s multiple comparison test. Statistical significance was set at *p* < 0.05.

## Supplementary Materials

The following supporting information can be downloaded at: https://www.mdpi.com/article/10.3390/ijms23010213/s1.
