# Supplementary material for: Rigosertib and Cholangiocarcinoma: A Cell Cycle Affair
Source: Int J Mol Sci. 2021 Dec 25;23(1):213. doi: 10.3390/ijms23010213 (PMC8745771; doi:10.3390/ijms23010213)
Supplement: Supplementary file 1 [file ijms-23-00213-s001.zip › ijms-1512001-supplementary.pdf]

International Journal of
*Molecular Sciences*

MDPI

*Communication*

# Rigosertib and cholangiocarcinoma: a cell cycle affair

**Alessio Malacrida, Guido Cavaletti and Mariarosaria Miloso**

**Supplementary Figure**

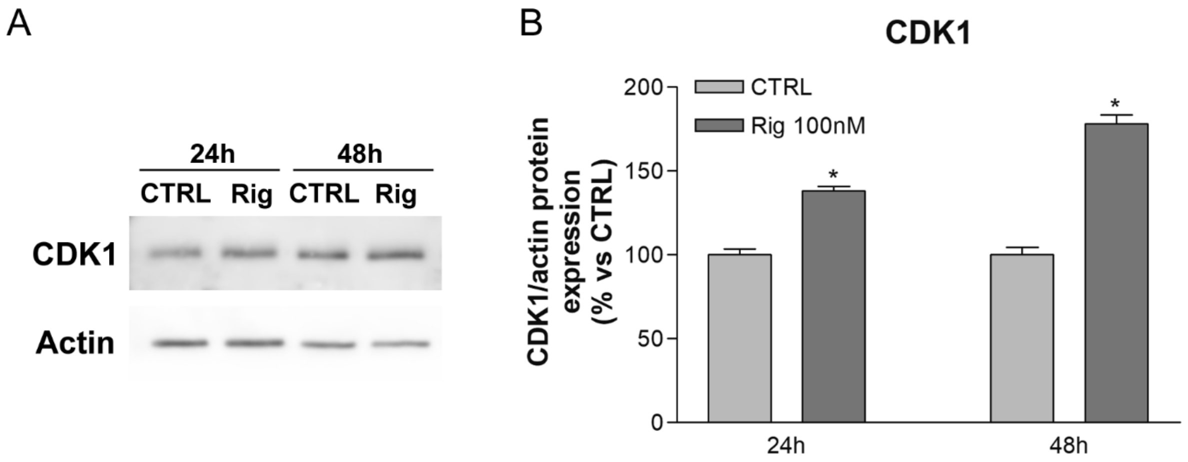


**Supplementary Figure S1.** Western blot analysis of CDK1 in EGI-1 cells treated with Rig. (**A**) Representative images of CDK1 and actin after treatment with 100 nM Rig for 24 and 48 h. (**B**) The graph represents the quantification of CDK1 western blot, normalized to actin. All the graphs are represented as the mean percentage ± SD of at least three independent experiments and are compared to untreated controls arbitrarily set to 100%. * $p < 0.05$ vs CTRL.
